# High-quality genome assembly and genetic mapping reveal a gene regulating flesh color in watermelon (*Citrullus lanatus*)

**DOI:** 10.3389/fpls.2023.1142856

**Published:** 2023-03-01

**Authors:** Hualin Nie, Moonkyo Kim, Sanghee Lee, Sohee Lim, Mi Sun Lee, Ju Hyeok Kim, Sol Ji Noh, Seong Won Park, Sang-Tae Kim, Ah-Young Shin, Yi Lee, Suk-Yoon Kwon

**Affiliations:** ^1^ Plant Systems Engineering Research Center, Korea Research Institute of Bioscience and Biotechnology, Daejeon, Republic of Korea; ^2^ Department of Industrial Plant Science and Technology, Chungbuk National University, Cheongju, Republic of Korea; ^3^ Division of Life Science, Korea Polar Research Institute, Incheon, Republic of Korea; ^4^ Biosystems and Bioengineering Program, Korea Research Institute of Bioscience and Biotechnology (KRIBB) School of Biotechnology, University of Science and Technology, Daejeon, Republic of Korea; ^5^ Watermelon and Strawberry Research Institute, Chungcheongbuk-do Agricultural Research and Extension Services, Cheongju, Republic of Korea; ^6^ Department of Medical and Biological Sciences, Catholic University of Korea, Bucheon, Republic of Korea; ^7^ Department of Bioinformatics, Korea Research Institute of Bioscience and Biotechnology (KRIBB) School of Bioscience, University of Science and Technology, Daejeon, Republic of Korea

**Keywords:** watermelon, genome assembly, linkage map, quantitative trait locus (QTL), carotenoid biosynthesis, phytoene synthase

## Abstract

The unique color and type characteristics of watermelon fruits are regulated by many molecular mechanisms. However, it still needs to be combined with more abundant genetic data to fine-tune the positioning. We assembled genomes of two Korean inbred watermelon lines (cv. 242-1 and 159-1) with unique color and fruit-type characteristics and identified 23,921 and 24,451 protein-coding genes in the two genomes, respectively. To obtain more precise results for further study, we resequenced one individual of each parental line and an F_2_ population composed of 87 individuals. This identified 1,539 single-nucleotide polymorphisms (SNPs) and 80 InDel markers that provided a high-density genetic linkage map with a total length of 3,036.9 cM. Quantitative trait locus mapping identified 15 QTLs for watermelon fruit quality-related traits, including β-carotene and lycopene content in fruit flesh, fruit shape index, skin thickness, flesh color, and rind color. By investigating the mapping intervals, we identified 33 candidate genes containing variants in the coding sequence. Among them, Cla97C01G008760 was annotated as a phytoene synthase with a single-nucleotide variant (A → G) in the first exon at 9,539,129 bp of chromosome 1 that resulted in the conversion of a lysine to glutamic acid, indicating that this gene might regulate flesh color changes at the protein level. These findings not only prove the importance of a phytoene synthase gene in pigmentation but also explain an important reason for the color change of watermelon flesh.

## Introduction

Watermelon (*Citrullus lanatus*, 2n = 2x = 22), originally from South Africa, is an edible fruit crop belonging to the family Cucurbitaceae (Bae et al., 2020) that is rich in lycopene, citrulline, vitamin C, and other essential micronutrients and vitamins (Dubey et al., 2021). Humans have domesticated and grown watermelons for 4,000 years (Paris, 2015). Although the original wild watermelon had small fruit (about 0.2 kg) with watery, hard-textured, pale-colored, and bland or bitter-tasting flesh, common watermelons have become sweet and savory after centuries of improvement (Paris, 2015; Guo et al., 2019; Chomicki et al., 2020). There are now more than 1,200 watermelon varieties worldwide with fruits of various sizes, shapes, rind patterns, and flesh colors (Perkins-Veazie et al, 2012; Porcher et al., 2013). According to the characteristics of their fruit, watermelons can be divided into three cultivar groups: *Citroides* (“Red-Seeded” preserving melon, red-seeded citron), *Lanatus* (Tsamma, Kalahari, South-African, and wild watermelons), and *Vulgaris* (commonly cultivated watermelon) (Guo et al., 2019).

The color of watermelon flesh is closely related to the carotenoid composition and can be defined as white, pale yellow, canary yellow, salmon yellow, orange, crimson red (red), scarlet red, or green (Zhao et al., 2013). In watermelons with red, pink, or scarlet flesh, lycopene accounts for 70%–90% of the total carotenoids (Perkins-Veazie et al., 2006; Nadeem et al., 2022). Watermelons with yellow flesh have high levels of xanthophylls (mainly neoxanthin, violaxanthin, and neochrome) (Bang et al., 2010; Liu et al., 2012). Watermelons with orange flesh have much more β-carotene, ζ-carotene, and prolycopene than other pigments (Tadmor et al., 2005). White-flesh watermelons have almost no pigment and only contain trace amounts of phytofluene (Wang et al., 2019). These differences in carotenoid composition are likely due to molecular variation in the processes of carotenoid biosynthesis and accumulation. In addition to flesh colors, a variety of watermelon rind colors are highly desirable to consumers, and breeders place considerable emphasis on this trait. The rind coloration of watermelon is affected by chlorophylls and carotenoids that are synthesized in plant plastids from metabolic precursors provided by the methylerythritol 4-phosphate (MEP) pathway (Simpson et al., 2016; Liu et al., 2020; Ma et al., 2021). The rind colors are likely to be influenced by the same or similar molecular mechanisms that influence the flesh colors (Liu et al., 2020; Xiao et al., 2022). Other traits that are important for watermelon quality include the shape, weight, firmness, and diameter of the fruit. Many previous studies have applied genetic mapping and gene prediction to investigate these unique traits in watermelons (Sandlin et al., 2012; Guo et al., 2015; Liu et al., 2020; Sun et al., 2020).

Several watermelon genomes have been assembled for functional genomics studies and genetic improvement. A non-bitter, white-fleshed Sudanese Kordofan melon (*C. lanatus* subsp. *Cordophanus*) assembled in 2019 appears to be the most closely related ancestor of the domesticated watermelon (Renner et al., 2021). The first high-quality assembled genome sequence of domesticated watermelon was released in 2013 as a result of comprehensive genomic and transcriptomic analyses of the East Asian watermelon cultivar 97103, which contains preferentially selected genomic regions and has lost many disease-resistant genes during domestication (Guo et al., 2013). Subsequently, Wu et al. (2019) created a high-quality genome assembly of a principal American cultivar, Charleston Gray, and conducted a comparative analysis of the Charleston Gray and 97103 genomes. Deng et al. (2022) recently completed assembly of the telomere-to-telomere gap-free genome G42 using high-coverage and accurate long-read sequencing data. These genomic resources were developed to study the evolution, domestication, and genotyping of watermelons. In addition, resequencing analyses have further improved capabilities to identify single-nucleotide polymorphisms (SNPs), simple sequence repeats, and InDels that can be used as co-dominant markers to determine the genotypes of watermelon populations (Rhee et al., 2015).

Watermelon cultivars 242-1 and 159-1 have been bred in the Chungcheongbuk-do Agricultural Research and Extension Services (Cheongju, Korea), which have unique characteristics of color and fruit type: 242-1 has orange flesh, black rind, and oval shape, whereas 159-1 has red flesh, yellow rind, and round shape. To provide more genetic information about watermelon cultivars with unique flesh colors, we assembled the genomes of cultivars 242-1 and 159-1 using Nanopore long-read sequencing and Illumina short-read sequencing. We then resequenced the whole genomes of F_2_ **population** to construct a high-density genetic map and perform quantitative trait locus (QTL) analysis. Finally, we investigated the mapped QTL regions to identify candidate genes associated with flesh quality-related traits, including lycopene, β-carotene, flesh color, skin color, skin thickness, and fruit shape index. Our results provide a basis for candidate gene selection, annotation, and functional verification to elucidate the genetics of valuable traits and contribute to the development of breeding strategies based on molecular markers.

## Materials and methods

### Plant materials and DNA sequencing

**Figure 1:**
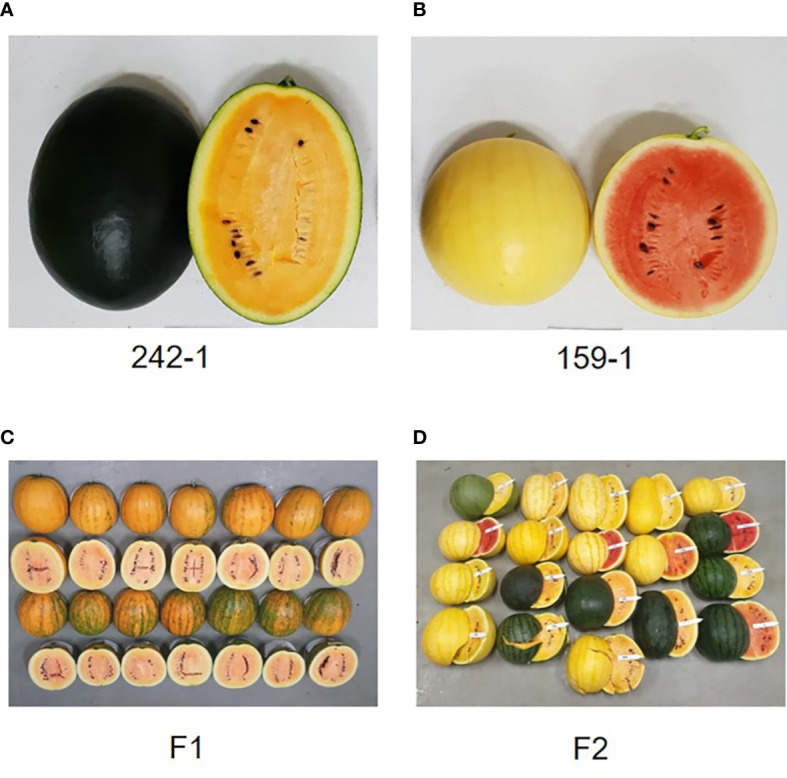
Morphological characteristics of the inbred parental lines and F_1_ and F_2_ individuals. **(A, B)** Fruits of parental cultivars 242-1 **(A)** and 159-1 **(B)**. **(C)** Fruits of F_1_ hybrids are round with striped rind and yellow flesh color. **(D)** Fruits of F_2_ individuals produced by self-pollination of F_1_ individuals. The F_2_ generation segregated into various rind colors (dark green, black, and mixture of black and dark green) and flesh colors (red, orange, and mixture of red and orange).

We selected the Korean inbred watermelon cultivars 242-1 and 159-1 as experimental materials because of their differential characteristics (**Figure 1**). Seeds of the 242-1 and 159-1 cultivars were kindly provided by Chungcheongbuk-do Agricultural Research and Extension Services (ASES; Cheongju, Korea). For DNA sequencing, seeds were planted in the ASES experimental field and harvested on day 80 of cultivation.

For genome assembly, high–molecular weight genomic DNA was isolated from the fresh young leaf tissues of 242-1 and 159-1 plants by performing a nuclei isolation step according to previous protocols (Zerpa-Catanho et al., 2021). Libraries for long-read sequencing were prepared by end-repair and dA-tailing, ligation, and purified-ligated DNA purification using NEBNext^®^ Ultra™ II End Repair/dA-Tailing Module (cat. no. E7546, New England Biolabs, Ipswich, MA, USA), NEBNext^®^ Quick Ligation Module (cat. no. E6056, New England Biolabs), and Ligation Sequencing Kit [cat. no. SQK-LSK109, Oxford Nanopore Technologies Co. (ONT), Oxfordshire, UK], respectively, according to recommendations by ONT. MinION sequencing was performed as per the manufacturer’s guidelines using R9.4 SpotON Flow Cell (cat. no. FLO-MIN106) and controlled using ONT MinKOW software (ONT). For short-read sequencing, genomic DNA paired-end (PE) and mate-pair (MP) libraries were constructed with 350–800, 550–800, and 600–800 bp insert sizes using the NEBNext Ultra DNA Library Prep Kit (New England Biolabs) and with 2- and 5-kb insert sizes using the Nextera Mate Pair Kit (Illumina, San Diego, CA, USA), respectively. The PE and MP libraries were sequenced at DNACARE Co. (Seoul, Korea)

### Genome assembly and pseudomolecule construction

We used the Trimmomatic v0.38 tool to check the quality of the Nanopore long-read sequences and delete the adapters, low-quality reads (reads that contained “N” as more than 10% of the nucleotides), and duplicated reads. The clean PE reads were assembled using the NextDenovo v2.5.0 software with the default parameters. The reads were mapped to an initial assembly using Minimap2 v2.17 with the options “–MD -ax map-ont -t 20 -L” and then sorted using SAMtools v1.16.1 software (Li et al., 2009). The raw assembly was polished with the short-reads from Illumina sequencing in three rounds using NextPolish v1.4.0 software (Hu et al., 2020). Then, haplotigs were removed using PurgeHaplotigs (Roach et al., 2018) with the default parameters. Finally, we validated the genome assemblies using the Benchmarking Universal Single-Copy Ortholog (BUSCO) v4.1.4 software (Simão et al., 2015) with 1,614 Nb of BUSCO markers in Embryophyta (odb10).

The assembled genome sequences were separated into chromosomal units corresponding to the reference sequence for *C. lanatus* cultivar 97103 (http://cucurbitgenomics.org/organism/1 ) using the NUCMER 4 program (Marçais et al., 2018). Anchoring was performed to determine the order and orientation of sequences within the chromosome. Pseudomolecules were constructed using an in-house perl script, and 100 N’s were artificially inserted between contigs to complete the sequence of each chromosome unit. The assembled pseudomolecules were then used for nucmer alignment with the 97103 reference genome, and a dot plot was generated using mummerplot to finally confirm the completeness of the pseudomolecules.

### Genome annotation, gene prediction, and functional annotation

The process of genome annotation was divided into three parts: repeat annotation, structural annotation, and functional annotation (**Supplementary Figure S1**). Repeat annotation was performed using a homology-based method and a *de novo* method in parallel. Homology-based repeat annotation was performed using RepeatMasker v4.0.3 (Tarailo-Graovac and Chen, 2009) with the plant repeat database PlantsRep (http://www.plantrep.cn/ ) (Luo et al., 2022). For *de novo* repeat annotation, RepeatModeler v1.0.8 (Flynn et al., 2020) was used to predict *de novo* repeats from the assembled genome sequence and construct a RepeatMasker library for further analysis. The results of the two repeat analyses were integrated, and the repetitive regions in the genome sequences were masked using bedtools maskfasta (Quinlan and Hall, 2010).

We downloaded and analyzed the Illumina short-read transcripts of five tissues (flesh, SRR10389406; leaf, SRR3156561; light green rind, SRR10803496; root, SRR12079410; and shoot apex, SRR3156569) of watermelon (*Citrullus lanantus*) from NCBI SRA database (https://www.ncbi.nlm.nih.gov/sra ) (Wang et al., 2021). We trimmed the raw RNA sequence using the trimmomatic v0.39 to obtain transcript sequences with an average phred quality score of at least 30 and a minimum length of 50 bp. By removing the Illumina adapter sequences, a total of 27 Gb of paired high-quality nucleotide sequences were obtained for annotation. Gene model predictions for structural annotation were made using the PASA v2.5.1 program (Haas et al, 2003) based on *de novo* transcript assemblies generated with the Trinity v2.8.6 program (Haas et al, 2013) and mapped-region information for RNA-seq short-read data generated with the StringTie v2.0.4 program (Kovaka et al., 2019). In addition, 22,596 protein sequences from the 97103 reference genome were mapped by the protein2genome method in the Exonerate v2.2.0 program and used for gene prediction (Slater and Birney, 2005). Finally, all prediction results (*ab initio*, transcript-based, and protein alignment) were merged to generate a non-redundant original gene set in Evidence Modeler (Haas et al., 2008).

Functional annotation of the gene set was performed by querying the protein sequences against the RefSeq (4,416,162 sequences; https://www.ncbi.nlm.nih.gov/refseq/ ), UniProt (557,491 sequences; https://www.uniprot.org ), and The Arabidopsis Information Resource (TAIR) (48,359 sequences; https://www.arabidopsis.org ) databases with an e-value cutoff of 1e−4. Analyses of conserved protein domains, gene ontology, and pathways were performed using the InterProScan program (Jones et al., 2014) and the Pfam, Gene Ontology, and Kyoto Encyclopedia of Genes and Genomes (KEGG) databases. Gene expression information was obtained for regions with FPKM values greater than 0.5 in the integrated gene model based on the watermelon RNA-seq maps.

### Fruit phenotype analysis

The 242-1 and 159-1 watermelon cultivars were used as maternal and paternal lines, respectively, to produce F_1_ and F_2_ progeny. The F_1_ hybrid was produced by the crossing cultivars 242-1 and 159-1. The F_2_ plants were subsequently produced by self-pollination of an F_1_ plant and used for whole-genome sequencing analysis, genetic linkage mapping, QTL mapping, and phenotyping of fruit-related traits.

The β-carotene and lycopene of F_2_ individuals were extracted from freeze-dried watermelon flesh according to the method described by Lee et al. (2022). Briefly, 0.1 g of finely crushed watermelon sample was added to 1 ml of ethanol containing 0.5 mM butylated hydroxytoluene and mixed carefully. Then, 3 ml of petroleum ether was added and vortexed, followed by 8 ml of 20% NaCl and additional vortexing for 1 min. After centrifugation at 3,000 rpm for 10 min, the supernatant was collected, and 1:1 (v/v) Na_2_SO_4_ was added and mixed. Finally, the sample was filtered with a 13-mm 0.2-μm Polytetrafluoroethylene (PTFE) Syringe Filter (Advantec Co., Tokyo, Japan) for analysis. Lycopene content was determined with an UltiMate 3000RS High performance liquid chromatography (HPLC) system (Thermo Fisher Scientific Inc.) equipped with a Kinetex 2.6-µm C18 100A reversed-phase column 100 × 4.60 mm (Phenomenex, Torrance, CA, USA), using 78% methanol for phase A and 100% ethyl acetate for phase B at a flow rate of 1 ml/min. The separation conditions were as follows: 0–8 min, 70% solution B; 8–10 min, 60% solution B; 10–12 min, 100% solution B; 12–14.01 min, 0% solution B; and 14.01–20 min, 100% solution B. Trans-lycopene (Sigma-Aldrich, St. Louis, MO, USA) and β-carotene (Sigma-Aldrich) were used as quantitative standards. Lycopene and β-carotene were quantified in the samples by measuring absorbance at 450 and 660 nm. The flesh color and rind color were expressed as red, green, and blue values using a Red-Green-Blue image analysis system (Aït-Aissa et al., 2018). Descriptive statistics and other statistical analyses were evaluated using SPSS v18.0 software (SPSS Inc., Chicago, IL, USA).

### Whole-genome sequencing of F_2_ populations and raw variant calling

For whole-genome resequencing, total genomic DNA was isolated from fresh young leaf tissues of F_2_ plants and two parental plants (242-1 and 159-1) using the DNeasy Mini Kit (QIAGEN, Hilden, Germany). DNA samples were combined using different barcode adapters (5 μl each) and purified using the QIAquick PCR Purification Kit (cat. no. 28104, QIAGEN) according to the manufacturer’s instructions. A 150-bp PE library with an insert size of 350 bp was constructed for each individual plant using the NEBNext Ultra DNA Library Prep Kit (New England Biolabs). The qualified libraries were sequenced using an Illumina Hi Seq 2500 platform to produce 150-bp PE reads. Quality trimming was performed with the sliding window (4:20), average quality (20), and minimum read size (36) options in the Trimmomatic v 0.38 program. The trimmed reads were aligned to the 242-1 genome (female) using BWA-MEM aligner. After correcting the map file information using Picard and GATK codebases, a variant call was performed to generate SNP and InDel information for each sample. To select high-quality SNPs, variants with biallelic extraction, depth of coverage < 10, genotype quality < 30, or missing rate < 10% were removed. A total of 36,632 contigs were created for the 242-1 cultivar, and candidate InDel loci were compared to corresponding loci in the 159-1 cultivar. We selected InDel loci with a variant ratio > 0.8, InDel size > 30 bp, and number of reads > 20 for more accurate genotyping.

### High-resolution mapping and QTL analysis of qualitative traits

A genetic map was constructed using the IciMapping v4.2 software (Institute of Crop Science Chinese Academy of Agricultural Sciences, Beijing, China) according to the following process: binning to remove duplicate markers, placing markers in linkage groups, ordering markers within linkage groups, and adjusting marker intervals within linkage groups (Meng et al., 2015). Markers were first filtered out by comparison of the offspring genotype distribution with the expected Mendelian proportions (1:2:1). A default value of data tolerance based on the X^2^ test (*p* < 0.05) was used to discard highly separated markers. Then, markers were grouped using a maximum threshold recombination fraction value of 0.3. The recombination frequency was converted to map distance by Kosambi mapping (Kosambi, 2016). QTL analysis was performed using the IciMapping v4.2 software (Meng et al., 2015) with the inclusive composite interval mapping method. The logarithm of the odds (LOD) threshold was analyzed by the permutation method with 1,000 repeats to limit the type I error to 0.05 or less.

## Results

### Genome sequencing and assembly

We assembled and annotated a complete genome for Korean inbred watermelon cultivars 242-1 and 159-1 (**Supplementary Figure S1**). Nanopore long-read sequencing and Illumina short-read sequencing generated 7.7 Gb (22× coverage) and 8.6 Gb (25× coverage) long-read sequences and 15.6 Gb (44.6× coverage) and 14.1 Gb (40.3× coverage) high-quality trimmed short-read sequences for the 242-1 and 159-1 cultivars, respectively (**Supplementary Table S1**). The assembled long-read sequences of cultivars 242-1 and 159-1 had total lengths of 361.7 and 362.1 Mb, containing 43 and 103 initial contigs with N50 lengths of 16.0 and 6.3 Mb, respectively (**Table 1**). Pseudomolecules were constructed at the chromosome level based on the *C. lanatus* 97103 genome. Initial contigs were assembled *de novo* and clustered into 11 chromosomes ranging in length from 24.4 to 37.8 Mb for the 242-1 cultivar and 26.7 Mb to 37.9 Mb for the 159-1 cultivar (**Supplementary Table S2**). The lengths of most of the assembled chromosomes were very similar between 242-1 and 159-1, except that chromosome 4 of cultivar 242-1 was 24.4 Mb, which was about 2.7 Mb shorter than that of *C. lanatus* 97103 and about 2.3 Mb shorter than that of cultivar 159-1. We also used long-read sequencing to assemble the chloroplast genomes of cultivars 242-1 and 159-1, which provided new usable genetic information (**Supplementary Figure S2**).

**Table 1:** Comparison of the two assembled genomes (242-1 and 159-1) with two reference watermelon genomes (Charleston Gray and 97103).

Quality metric	Charleston Gray	97103	242-1	159-1
Total sequence length (bp)	397,829,775	365,450,462	361,732,851	362,131,170
Number of contigs	20,073	358	43	103
Contig N50 (bp)	48,885	2,312,425	16,071,105	6,343,009
Number of scaffolds	6,794	113	N/A	N/A
Scaffold N50 (bp)	33,982,080	35,099,344	N/A	N/A
Number of genes	22,546	23,440	23,921	24,451

N/A, Not applicable.

**Figure 2:**
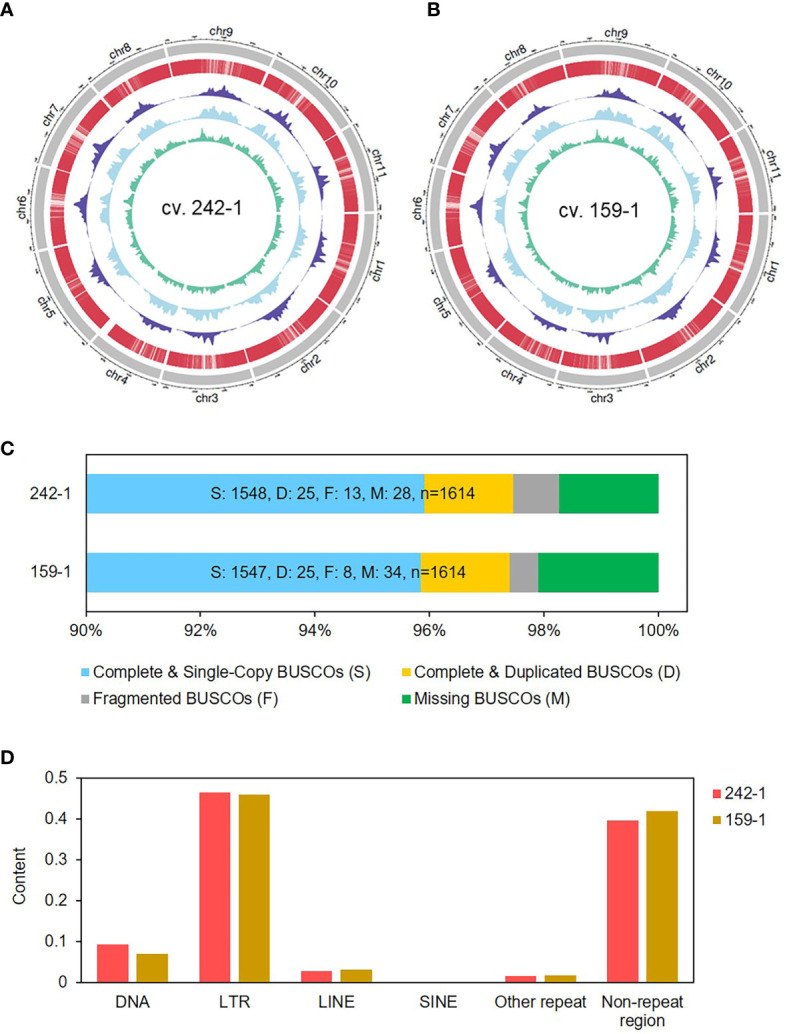
Genome characterization of the two inbred parental watermelon lines. **(A, B)** The outermost circle is the ideogram of 11 chromosomes in Mb scale, enclosing concentric circles of gene distribution (red), Gypsy content (navy blue), Copia content (sky blue), and DNA transposon content (green) in 1-Mb scale. **(C)** BUSCOs of the assembled watermelon genomes analyzed by using BUSCO v4.1.4 software. **(D)** Repetitive contents of the watermelon genomes analyzed by Repeat Masker with Repbase.

We assessed the completeness of the genome assemblies by aligning published RNA-seq reads and performing BUSCO analysis. The full-length Illumina short-read transcripts of five watermelon tissues (flesh, SRR10389406; leaf, SRR3156561; light green rind, SRR10803496; root, SRR12079410; and shoot apex, SRR3156569) were aligned to the genome sequences of 242-1 and 159-1 (Wang et al., 2021). The results showed that more than 83.2% of the full-length transcripts could be mapped to the retained reads (**Supplementary Table S3**). A total of 1,548 (95.9%) and 1,547 (95.8%) BUSCOs were detected in the assembled genome of cultivars 242-1 and 159-1, respectively, indicating equivalent assembly quality compared to other assembled watermelon genomes (**Figure 2C**; **Supplementary Table S4**). The assembled genomes had similar chromosome synteny with the 97103 reference genome, which was expected given that the 242-1 and 159-1 pseudochromosomes were generated according to the genes and sequence of the 97103 genome. We found a high number of syntenic blocks in most of the chromosomes, except for chromosome 4. The syntenic blocks between the 242-1 assembly and the 97103 genome had different patterns in chromosome 4 (**Supplementary Figure S3**). Whole-genome nucleotide alignment showed that both assembled genomes aligned closely with the 97103 reference genome (**Supplementary Figure S4**, **S5**).

### Repeat sequence annotation and gene prediction

We mapped the distributions of protein-coding genes, long terminal repeat (LTR)/Gypsy and LTR/Copia retrotransposons, and DNA transposons in the genome 242-1 and 159-1 to obtain an overview of the genome organization (**Figures 2A, B** **)**. Analysis of repeat sequences revealed that the 242-1 and 159-1 assemblies contained 206 Mb (57.6%) and 207 Mb (57.5%) of repeat sequences and only 87 Mb (24.0%) and 86 Mb (24.2%) of assigned genes and gene-related sequences, respectively (**Supplementary Figure S6**). LTR retrotransposons were the most abundant repeat elements, comprising 46.4% and 45.9% of the 242-1 and 159-1 assemblies, respectively, followed by DNA transposons (9.3% and 7.1%), long interspersed nuclear elements (3.0% and 3.2%; **Figure 2D**; **Supplementary Table S5**). Gene annotations of the 242-1 and 159-1 assemblies predicted 23,921 and 24,451 protein-coding genes, respectively (**Table 1**). Of those, 21,358 (89.3%) in the 242-1 assembly and 21,321 (87.2%) in the 159-1 assembly have putative functional descriptions in public databases (**Supplementary Table S6**).

### Carotenoid biosynthesis-related gene analysis

**Figure 3:**
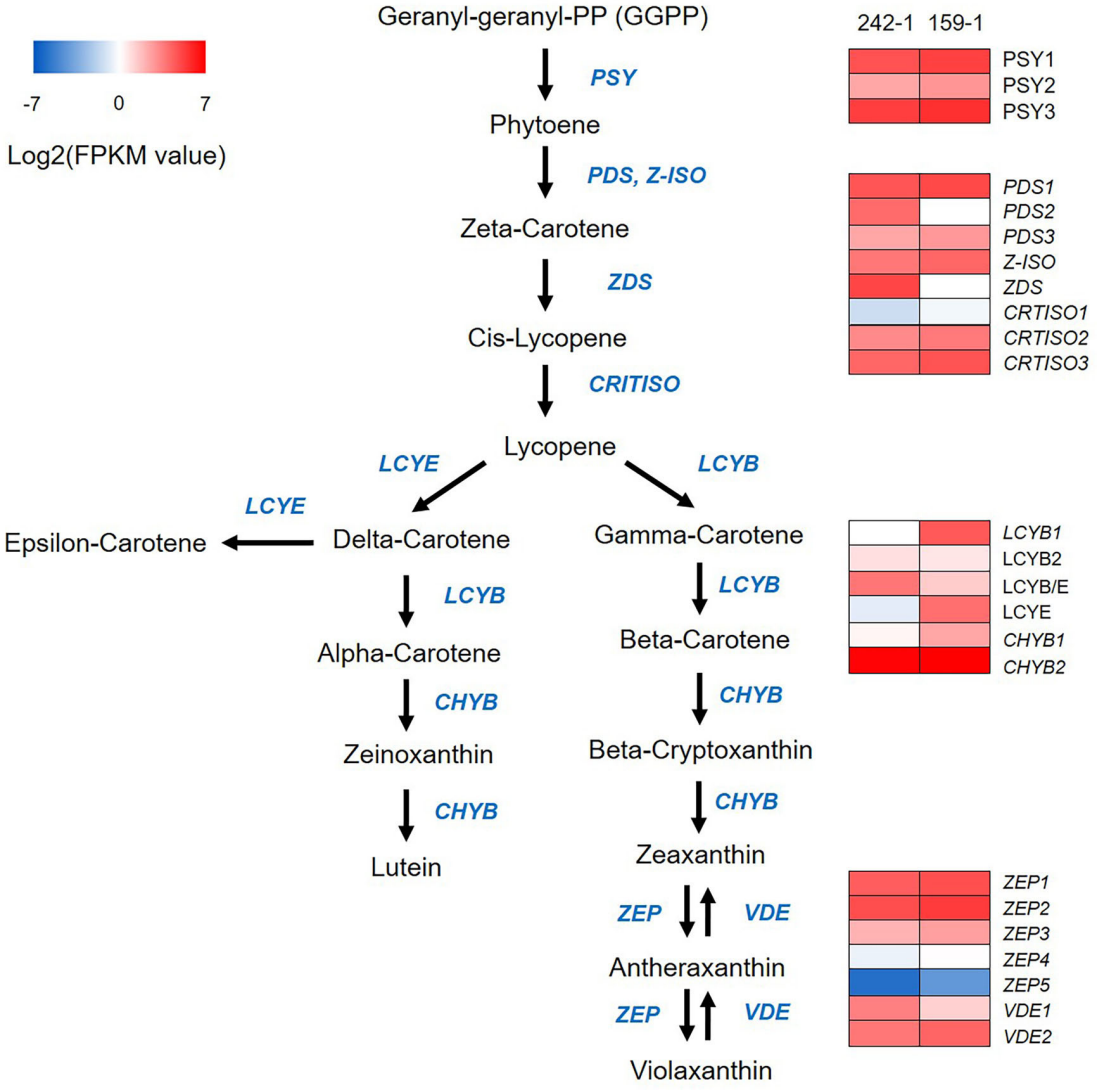
A heatmap diagram of expression levels of carotenoid biosynthetic pathway genes identified by KEGG analysis. The heatmap was drawn according to FPKM values calculated from Illumina short-read transcripts. Columns and rows in the heatmap represent samples and genes, respectively. Sample names are displayed above the heatmap. Color scale indicates fold changes in gene expression. PSY, phytoene synthase; PDS, phytoene desaturase; Z-ISO, zeta-carotene isomerase; CRTISO, beta-carotene isomerase; LCYB, lycopene beta-cyclase; LCYB/E, lycopene beta/epsilon cyclase; LCYE, lycopene epsilon cyclase; ZEP, zeaxanthin epoxidase; VDE, violaxanthin de-epoxidase.

We compared the expression of protein-coding genes involved in carotenoid biosynthesis between the 242-1 and 159-1 cultivars. Comparison of the Illumina short-read transcripts to annotated sequences in the KEGG database identified 24 genes in the carotenoid biosynthetic pathway that were expressed in 242-1 and 159-1: three phytoene synthases (*PSY1*, *PSY2*, and *PSY3*), three phytoene desaturases (*PDS1*, *PDS2*, and *PDS3*), one ζ-carotene isomerase (*Z-ISO*), one ζ-carotene desaturase (*ZDS*), three β-carotene isomerases (*CRTISO1*, *CRTISO2*, and *CRTISO3*), four lycopene cyclases (*LCYB1*, *LCYB2*, *LCYB/E*, and *LCYE*), two β-carotene hydroxylases (*CHYB1* and *CHYB2*), five zeaxanthin epoxidases (*ZEP1*, *ZEP2*, *ZEP3*, *ZEP4*, and *ZEP5*), and two violaxanthin de-epoxidases (*VDE1* and *VDE2*) (**Figure 3**; **Supplementary Table S7**). A heatmap of these genes based on FPKM values calculated from the Illumina short-read transcripts showed that upstream genes for phytoene synthesis (*PSY1*, *PSY2*, and *PSY3*) and the downstream genes for zeaxanthin epoxide synthesis (*ZEP1*, *ZEP2*, *ZEP3*, *ZEP4*, *ZEP5*, *VDE1*, and *VDE2*) in the carotenoid biosynthesis pathway had similar expression in the 242-1 and 159-1 cultivars. By contrast, most of the genes for lycopene synthesis (*PDS2*, *ZDS*, and *CRTISO1*) and carotene synthesis (*LCYB1*, *LCYB/E*, and *CHYB1*) had different expressions between 242-1 and 159-1.

### Genotyping and genetic linkage mapping

For genetic linkage mapping, a total of 1.1 Tb of WGS data comprising 7.5 billion reads were generated from the two parental samples (242-1 and 159-1) and the 87 F_2_ samples (**Supplementary Table S8**). A total of 2,029,598 raw SNPs and 869,298 InDels were identified among all individuals using VCF tools (Danecek et al., 2011). Of those, 686,357 SNPs and 937 InDels passed through quality filtering (see Materials and Methods; **Supplementary Table S9**). We selected 140,650 homozygous SNPs by screening for SNPs with the same genotype as the 242-1 (maternal) sequence (AA) and a different genotype than the 159-1 (paternal) sequence (BB). After the linkage maps were subjected to a binning process to remove duplicate markers, a total of 2,319 SNP markers were selected. In addition, a total of 167 InDels were selected and used to genotype the individual samples. These InDel markers were tested by PCR and electrophoresis analysis to confirm the genotypes of 242-1, 159-1, and F_1_ samples. As a result, 126 InDel markers were finally selected for genotyping of the F_2_ samples (**Supplementary Table S10**). On the basis of the electrophoresis bands of the selected InDel markers, each F_2_ individual was genotyped for each marker as maternal type, paternal type, or F_1_ type (heterozygous) (**Supplementary Figure S7**).

**Figure 4:**
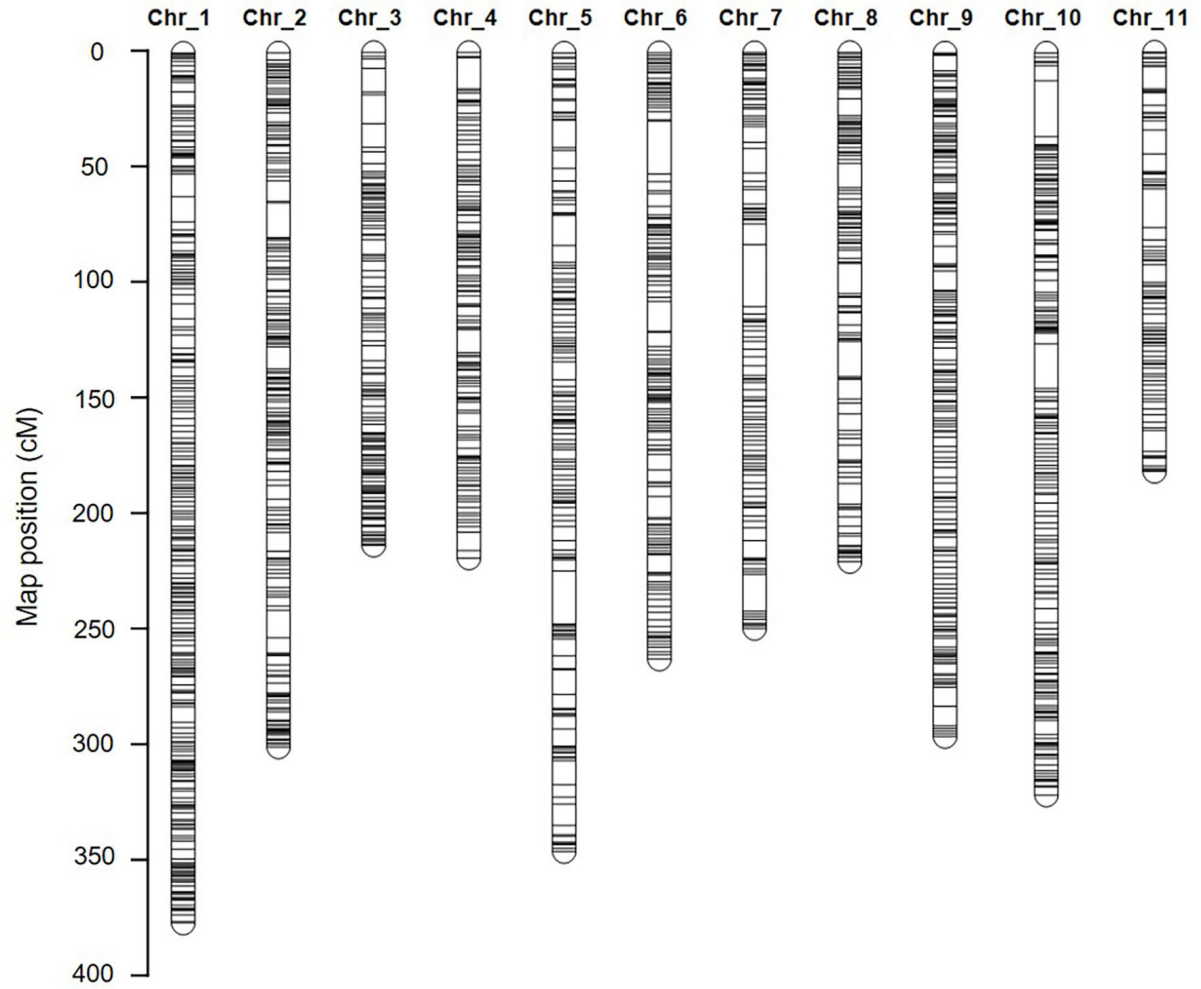
The high-density genetic linkage map of watermelon. The inter-specific linkage map of the inbred watermelon lines 242-1 and 159-1 harboring 1,619 loci. SNP and InDel markers are represented in black.

A total of 780 (33.6%) SNP markers and 45 (35.7%) InDel markers did not conform to a Mendelian ratio of 1:2:1 in the F_2_ samples and were discarded. As a result, a total of 1,619 SNP and InDel markers were identified and prepared for genetic linkage mapping (**Supplementary Tables S11**; **Table S12**). These markers were sorted into 11 linkage groups (**Figure 4**), which is consistent with the reported haploid chromosome number of watermelon (Guo et al., 2013). The genetic linkage map spanned 3,036.9 cM, with an average interval of 1.87 cM. The genetic length of each chromosome ranged from 187.37 cM (chromosome 11) to 379.57 cM (chromosome 01), with an average SNP distance of 1.56–2.48 cM (**Figure 4** and **Supplementary Table S11**).

### QTL mapping of fruit quality-related traits

Descriptive statistics for fruit quality-related traits are shown in **Table 2**; **Supplementary Table S13**. As the main components of flesh pigment, individuals in the F_2_ mapping family had lycopene levels ranging from 0.13 to 38.3 μg/g and β-carotene levels ranging from 0.17 to 5.24 μg/g. The flesh and rind color of the red, green, and blue values were obvious differences among F_2_ individuals. We performed Kolmogorov-Smirnov and Shapiro-Wilk tests (Razali and Wah, 2011) to investigate the normality of each fruit quality-related trait among the samples. The frequency distributions of the traits are shown in **Supplementary Figure S8**. Traits related to red or green color in the flesh or rind were strongly correlated with each other (r = 0.92 for flesh color, r = 0.99 for rind color, *P*-value < 0.01 for all). Traits related to lycopene content were also strongly correlated with traits related to red or green flesh color (r = −0.8 for red flesh, r = −0.84 for green flesh, *P*-value < 0.01 for all; **Supplementary Figure S9**). This result showed that the red or green flesh color were closely related to lycopene content in watermelon.

**Table 2:** Descriptive statistics of the traits investigated in the F_2_ population.

Trait	Mean ± SE	5% Trimmed mean	Median	Variance	Standard deviation	Minimum	Maximum	IQR	Skewness	Kurtosis
FSI	1.23 ± 0.02	1.2	1.13	0.06	0.24	0.94	2.0	0.24	1.41	1.34
SKT	0.92 ± 0.03	0.91	0.9	0.07	0.27	0.5	1.6	0.4	0.54	−0.26
L	8.80 ± 1.02	7.89	4.01	89.82	9.48	0.1	38.3	15.1	1.18	0.65
BC	2.02 ± 0.12	1.95	1.96	1.2	1.1	0.17	5.24	1.35	0.89	0.82
FR	220.09 ± 2.20	220.63	223	421.4	20.5	180	250	39	−0.33	−1.2
FG	139.02 ± 6.66	140.14	155	3860.9	62.1	27	225	118	−0.34	−1.38
FB	76.90 ± 3.85	75.73	74	1290	35.9	4	173	53	0.49	−0.24
RR	160.02 ± 9.41	162.62	226	7706.1	87.8	28	244	179	−0.46	−1.71
RG	141.24 ± 7.14	153.47	193	4411.5	66.4	36	230	125	−0.56	−1.45
RB	70.13 ± 5.00	68.61	60	2171.9	46.6	1	173	72	0.5	−0.8

IQR, interquartile range; FSI, fruit shape index; SKT, skin thickness (cm); L, lycopene (μg/g); BC, β-carotene (μg/g); FG, flesh color (green); FR, flesh color (red); RB, rind color (blue); RG, rind color (green); RR, skin color (red).

**Figure 5:**
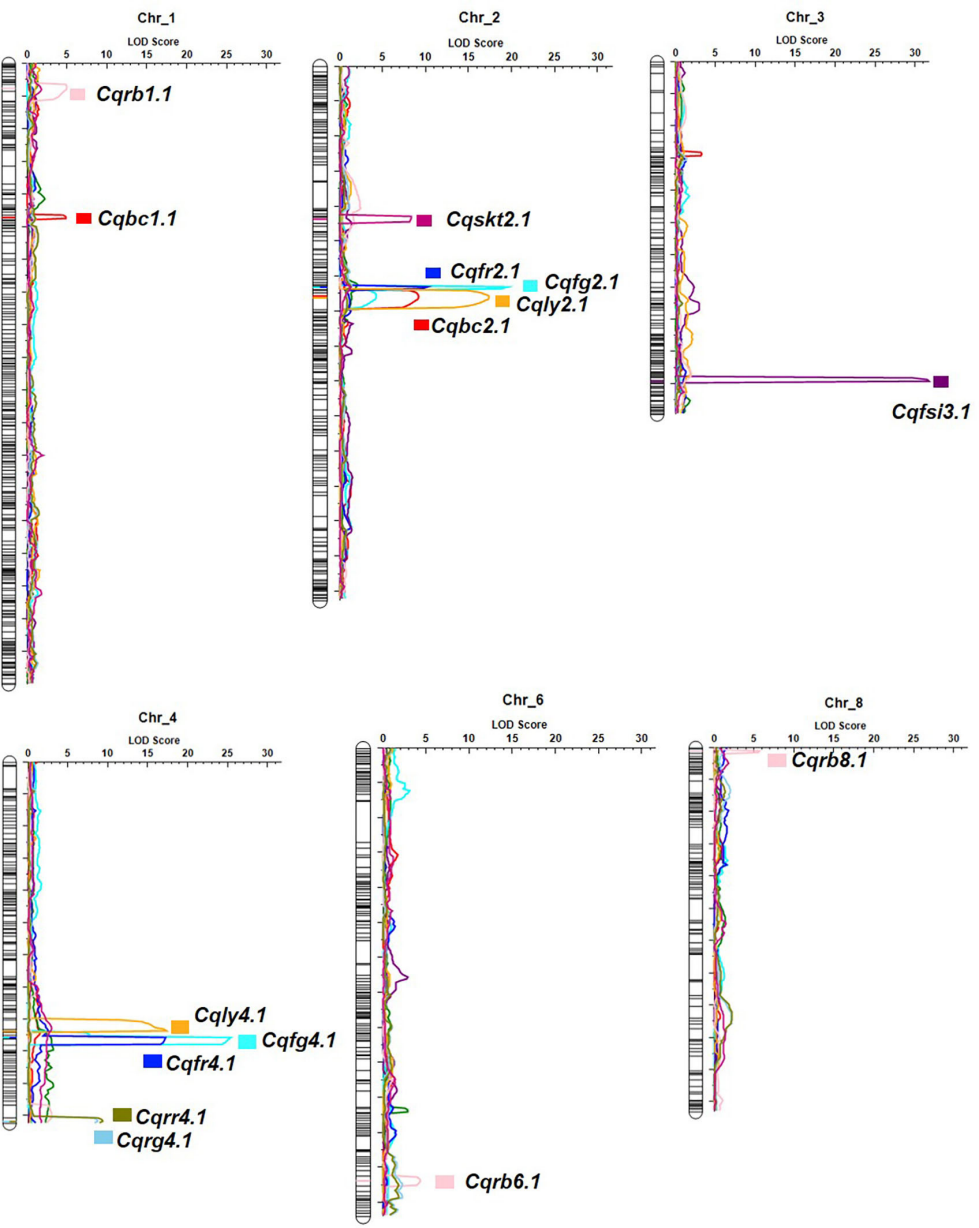
Genome-wide QTL mapping of fruit quality-related traits in the F_2_ population. The boxes on the right of the chromosomes represent QTLs for fruit shape index (Cqfsi3.1), skin thickness (Cqskt2.1), lycopene (Cqly2.1 and Cqly4.1), β-carotene (Cqbc1.1 and Cqbc2.1), flesh color (Cqfg2.1, Cqfg4.1, Cqfr2.1, and Cqfr4.1), and rind color (Cqrb1.1, Cqrb6.1, Cqrb8.1, Cqrg4.1, and Cqrr4.1).

Fifteen QTL intervals (one for fruit shape index, one for skin thickness, two for lycopene content, two for β-carotene content, four for flesh color, and five for skin color) associated with fruit quality-related traits were identified on chromosomes 1, 2, 3, 4, 6, and 8 by permutation tests (*p* < 0.05, LOD > 4.3; **Figure 5**; **Table 3**). The QTL interval with the highest LOD value of 25.4 was located at 171 cM between the markers ClaB_Chr04_14736633 and ClaB_Chr04_14817220 and accounted for 47.3% of the percentage variance explained (PVE). Eight QTL intervals contributed simultaneously to two traits: *Cqly2*.1 and *Cqbc2.1* to lycopene and β-carotene levels; *Cqfg2.1*, *Cqfr2.1*, *Cqfg4.1*, and *Cqfr4.1* to green and red fruit colors; and *Cqrg4.1* and *Cqrr4.1* to read and green skin colors.

**Table 3:** Characteristics of the fruit quality-related QTLs in the watermelon (LOD > 4.3).

QTL	Trait	Chr	Position (cM)	Marker interval	LOD	PVE (%)	Add	Dom	Left CI	Right CI
*Cqfsi3.1*	FSI	3	198	ClaB_Chr03_28459812–ClaB_Chr03_28508794	31.7	79.6	0.3	−0.17	196.5	198.5
*Cqskt2.1*	SKT	2	87	ClaB_Chr02_30520465–ClaB_Chr02_30520917	8.3	34.9	−0.2	0.0	85.5	89.5
*Cqly2.1*	L	2	132	ClaB_Chr02_26461833–ClaB_Chr02_25758590	17.3	36.0	−9.6	−6.6	129.5	135.5
*Cqly4.1*	L	4	167	ClaB_Chr04_16911823–Clg-InDel-165	17.4	32.5	−8.6	−6.2	165.5	167.5
*Cqbc1.1*	BC	1	94	ClaB_Chr01_9498243– ClaB_Chr01_9770736	4.9	15.7	59.7	−0.1	92.5	95.5
*Cqbc2.1*	BC	2	131	ClaB_Chr02_26461833–ClaB_Chr02_25758590	9.2	34.0	−86.9	−24.0	128.5	136.5
*Cqfg2.1*	FG	2	126	ClaB_Chr02_26728528–ClaB_Chr02_26706701	19.5	30.6	51.3	−0.4	125.5	127.5
*Cqfg4.1*	FG	4	171	ClaB_Chr04_14817220–ClaB_Chr04_14736633	25.4	47.3	64.3	−3.2	170.5	174.5
*Cqfr2.1*	FR	2	126	ClaB_Chr02_26728528–ClaB_Chr02_26706701	10.2	23.7	13.7	4.3	125.5	127.5
*Cqfr4.1*	FR	4	171	ClaB_Chr04_14817220–ClaB_Chr04_14736633	17.2	47.9	20.0	6.4	170.5	175.5
*Cqrb1.1*	RB	1	15	ClaB_Chr01_1527902–ClaB_Chr01_2118855	5.0	14.9	−4.2	−35.4	12.5	19.5
*Cqrb6.1*	RB	6	247	ClaB_Chr06_26810713–ClaB_Chr06_27345836	4.4	12.4	−7.2	30.7	244.5	250.5
*Cqrb8.1*	RB	8	2	ClaB_Chr08_27454491–ClaB_Chr08_27488871	5.7	17.0	−28.1	3.9	1.5	3.5
*Cqrg4.1*	RG	4	223	ClaB_Chr04_6127109–ClaB_Chr04_6095999	8.7	35.3	−54.7	21.1	220.5	224.0
*Cqrr4.1*	RR	4	223	ClaB_Chr04_6127109–ClaB_Chr04_6095999	9.3	37.3	−73.8	30.2	220.5	224.0

PVE, percentage variance explained; Add, QTL with additive effect only; Dom, QTL with complete dominance; CI, confidence interval (cM); FSI, fruit shape index; SKT, skin thickness; L, lycopene; BC, β-carotene; FG, flesh color (green); FR, flesh color (red); RB, rind color (blue); RG, rind color (green); RR, skin color (red).

**Figure 6:**
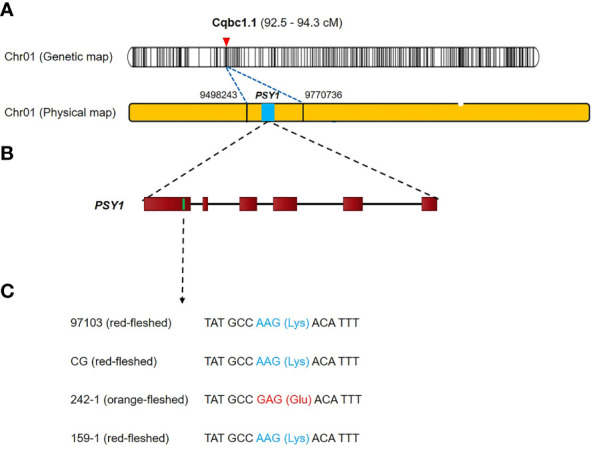
Genetic mapping and phenotypic analysis of the carotenoid biosynthesis gene *PSY1* in watermelon. **(A)** The preliminary genetic map and physical map. The *PSY1* gene is located on chromosome 1. **(B)** Structure of the *PSY1* gene. A nonsynonymous SNP mutant is located in the first exon at 9,539,129 bp on chromosome 1. **(C)** The SNP (A → G) results in the conversion of a lysine (Lys) to a glutamic acid (Glu).

We used BLAST to search for gene sequences in the 15 fruit quality-related QTL intervals based on the annotation of the 97103 reference genome in the Cucurbit Genomics Database (http://cucurbitgenomics.org/blast ) (Zheng et al., 2019). We identified a total of 302 candidate genes in the 15 QTL intervals. The QTL interval with the most candidate genes was *Cqrb6.1*, which contained 67 candidate genes, followed by *Cqrb1.1* with 66 candidate genes, whereas *Cqskt2.1*, *Cqrg4.1*, and *Cqrr4.1* each contained one candidate gene (**Supplementary Table S14**). We compared the candidate genes between the parental cultivars using the resequencing data and identified 33 genes that had variation in an exon (**Supplementary Table S15**). We then performed gene ontology-enrichment analysis and KEGG pathway analysis to investigate the functions of these 33 genes. The results identified two genes involved in carotenoid biosynthesis that were also linked to flesh color, phytoene synthase 1 (*PSY1*, *Cla97C01G008760*), and red chlorophyll catabolite reductase (*RCCR*, *Cla97C02G03827*0), which were located in the QTL intervals *Cqbc1.1* and *Cqly2.1*, respectively (**Supplementary Table S15**)*. RCCR* had a single-nucleotide variant (C → G) in the first exon at the 25,790,540 bp of chromosome 2 that resulted in conversion of an asparagine to a lysine. *PSY1* had a single-nucleotide variant (A → G) in the first exon at 9,539,129 bp of chromosome 1 that resulted in conversion of a lysine to glutamic acid (**Figure 6**). In 242-1 and 159-1, the *PSY1* variant encoding the glutamic acid was exclusive to 242-1, which was the only cultivar with non-red flesh (orange flesh).

## Discussion

The three previously assembled domesticated watermelon genomes (Charleston Gray, 97103, and G42) are all from cultivars with green skin and red flesh (Guo et al., 2013; Wu et al., 2019; Deng et al., 2022). Our genome assemblies provide new information on watermelon cultivars with black or yellow skin and orange flesh. Chromosome 4 of the red-flesh, yellow-rind 159-1 cultivar is similar to that of the red-flesh 97103 reference cultivar, whereas chromosome 4 of the orange-flesh, black-rind 242-1 cultivar has a different construction. There are many genes and regions associated with fruit color (Guo et al., 2019; Wang et al., 2019; Wang et al., 2021) and rind color (Park et al., 2016; Dou et al., 2018; Liu et al., 2020) on chromosome 4, which indicates the specificity of the 242-1 genome. The distinguishing characteristics of the 242-1 and 159-1 watermelon genomes are mainly reflected in flesh and rind colors. The carotenoid biosynthesis-related genes identified in the two new assembled genomes provide a basis for the mapping of watermelon fruit color and rind color.

The color of watermelon flesh is mainly linked to the composition of xanthophylls and carotenes (mainly phytoene, lycopene, and β-carotene) (Wang et al., 2021). We found that two lycopene biosynthesis-related genes, *PDS2* and *ZDS*, had higher expression in the orange-fleshed 242-1 cultivar than in the red-fleshed 159-1 cultivar. The *PDS*, *ZDS*, and *CRTISO* genes are considered to play only connecting roles in lycopene biosynthesis and accumulation (Kato et al., 2004). These results suggest that *PDS2* catalysis of ζ-carotene biosynthesis and *ZDS* catalysis of cis-lycopene biosynthesis are more active in orange-fleshed watermelon (242-1) than in red-fleshed watermelon (159-1), but this difference does not result in a change of lycopene content. The expression of lycopene cyclase genes (*LCYB1*, *LCYB/E*, and *LCYE*) that regulate α-carotene, β-carotene, γ-carotene, δ-carotene, and ϵ-carotene biosynthesis from lycopene was also different between the 242-1 and 159-1 cultivars, suggesting that the two watermelon cultivars have very different mechanisms of carotene synthesis and accumulation.

A single-nucleotide variant of the *PSY1* (*Cla97C01G008760*) gene located in the β-carotene–mapping QTL interval *Cqbc1.1* differs between the orange-fleshed cultivar 242-1 and the three red-fleshed cultivars 97103, Charleston Gray, and 159-1, suggesting that this variant has a strong impact on flesh color. Phytoene synthase is the first enzyme in the carotenoid biosynthesis pathway and converts geranylgeranyl diphosphate into phytoene. This enzyme defines the size of the carotenoid pool, which suggests that the variation that we detected in *PSY1* affects the carotenoid composition of the watermelon fruit, resulting in the orange color in cultivar 242-1. Orange-flesh watermelon is a landrace of red-flesh watermelon, and coral-red flesh (*Y*) is dominant to orange flesh (*y°*) (Bang et al., 2010; Guo et al., 2019). This implies that red-flesh watermelon evolved from orange-flesh watermelon during a process of selective breeding and domestication, during which variation of *PSY1* arose and resulted in a decrease in β-carotene content and an increase in lycopene content. On the basis of QTL mapping of variants related to lycopene and β-carotene synthesis, we found an *RCCR* gene in the QTL intervals *Cqly2.1* and *Cqbc2.1*. RCCR is essential in chlorophyll degradation during leaf senescence and fruit ripening in higher plants (Hörtensteiner, 2006), and this chlorophyll degradation is closely linked to the formation of carotenoids (Meier et al., 2011; Tripathy and Pattanayak, 2012; Leng et al., 2017). Our results suggest that the *RCCR* gene participates in the regulation of lycopene and β-carotene biosynthesis and accumulation in watermelon fruit. A previous study reported that levels of various carotenoids, such as lutein, β-carotene, violaxanthin, and zeaxanthin, were highly increased in *RCCR*-silenced tobacco plants (Dong et al., 2022). Furthermore, although lycopene was not mentioned in that report, expression of the phytoene synthase *NbPSY1* and the β-carotene isomerase *NbCRTISO*, both key genes in lycopene synthesis, was also increased after *RCCR* silencing (Dong et al., 2022). This may be because there is no important mechanism for lycopene accumulation in tobacco leaves. In tomatoes, the lycopene and chlorophyll contents are inversely proportional, and lycopene synthesis and chlorophyll decomposition are synchronized (Carrillo-López and Yahia, 2014; Arthanari and Dhanapalan, 2019). Our results confirmed that *RCCR* plays an important role in controlling the chlorophyll and carotenoid contents in leaves and fruits of higher plants, although the mechanism varies among plants.

Like the flesh, the rind of watermelon fruit can have various colors, including black, dark green, light green, or yellow (Guo et al., 2013). Yellow-rind watermelon also has high carotenoid contents (Dou et al., 2018); however, there have been few genetic studies of the mechanism of rind color inheritance in watermelons. Previously, rind color-related loci were detected at the ends of chromosomes 4, 6, 8, and 10 (Jones et al, 2014; Park et al., 2016; Park et al., 2018). Genes involved in fruit skin color were previously linked to pigment binding; chloroplast membrane development; anthocyanin, porphyrin, and chlorophyll metabolism; hormone signal transduction; photosynthesis; and carotenoid biosynthesis (Hu et al, 2020; Liu et al., 2020). We found five rind color-related QTL intervals (Cqrb1.1, Cqrg4.1, Cqrr4.1, Cqrb6.1, and Cqrb8.1) on chromosomes 1, 4, 6, and 8, suggesting that rind color-related loci are located mainly on chromosomes 4, 6, and 8. We identified five candidate genes with CDS variation that might be involved in rind color (Cla97C04G070840, Cla97C06G124570, Cla97C06G124600, Cla97C06G124910, and Cla97C08G160580). In previous studies, the Dgo gene (Cla97C04G068530/Cla002769), which is involved in chlorophyll synthesis, was found to be involved in determining the background rind color (Park et al., 2016), and the ClCGMenG gene, which encodes 2-phytyl-1,4-beta-naphthoquinone methyltransferase, was found to be involved in determining dark green or light green color in the rind (Li et al., 2019). However, neither of these genes mapped to our candidate regions. Cultivars 242-1 and 159-1 have black and yellow rinds, respectively, which is different from the green-rind watermelons used in previous studies (Li et al., 2009; Park et al., 2016; Park et al., 2018; Li et al., 2019). The black and yellow rind colors may be the result of loci and candidate genes identified in our screen.

In summary, we performed genetic mapping of traits related to watermelon fruit quality, especially flesh color and rind color, and identified important loci and genes. A phytoene synthase encoded by *Cla97C01G008760* is likely to be a core element regulating the carotenoid metabolic pathways in the fruits of watermelon cultivars 242-1 and 159-1, which might have a chain effect on the downstream synthesis or accumulation of other substances, thereby changing the flesh color. We will continue to pay attention to this variation and use further genetic analysis to study the mechanisms that determine the color of watermelon flesh.

## Data availability statement

The data presented in the study are deposited in the National Center for Biotechnology Information (https://www.ncbi.nlm.nih.gov/) repository, accession number PRJNA924512 and PRJNA924516.

## Author contributions

S-YK and YL designed the project. SN and SP collected and grew the plant material. SLe, SLi, ML, and JK performed the experiments. MK performed the data analysis. HN conducted software analysis and wrote the original draft. S-TK, A-YS, YL, and S-YK revised the manuscript. All authors contributed to the article and approved the submitted version.
