# Supplementary material for: High-quality genome assembly and genetic mapping reveal a gene regulating flesh color in watermelon (Citrullus lanatus)
Source: Front Plant Sci. 2023 Mar 1;14:1142856. doi: 10.3389/fpls.2023.1142856 (PMC10014564; doi:10.3389/fpls.2023.1142856)
Supplement: Supplementary file 1 [file DataSheet_1.zip › Supplementary figures.pdf]

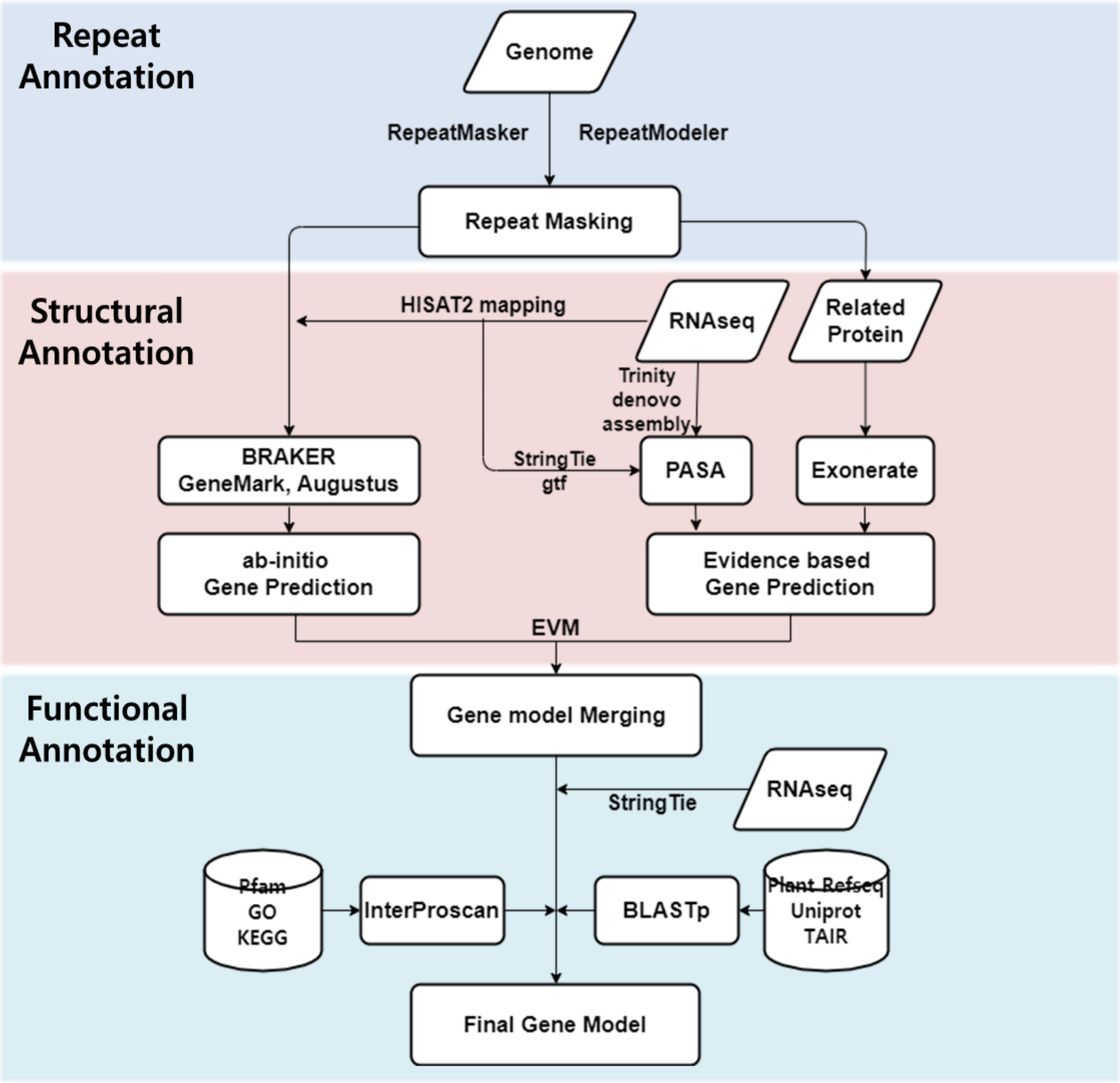

Supplementary Figure 1. Flowchart of the genome annotation. Genome annotation to predict the location and function of genes in the genome proceeds in three main steps: repeat annotation, structural annotation, and functional annotation.

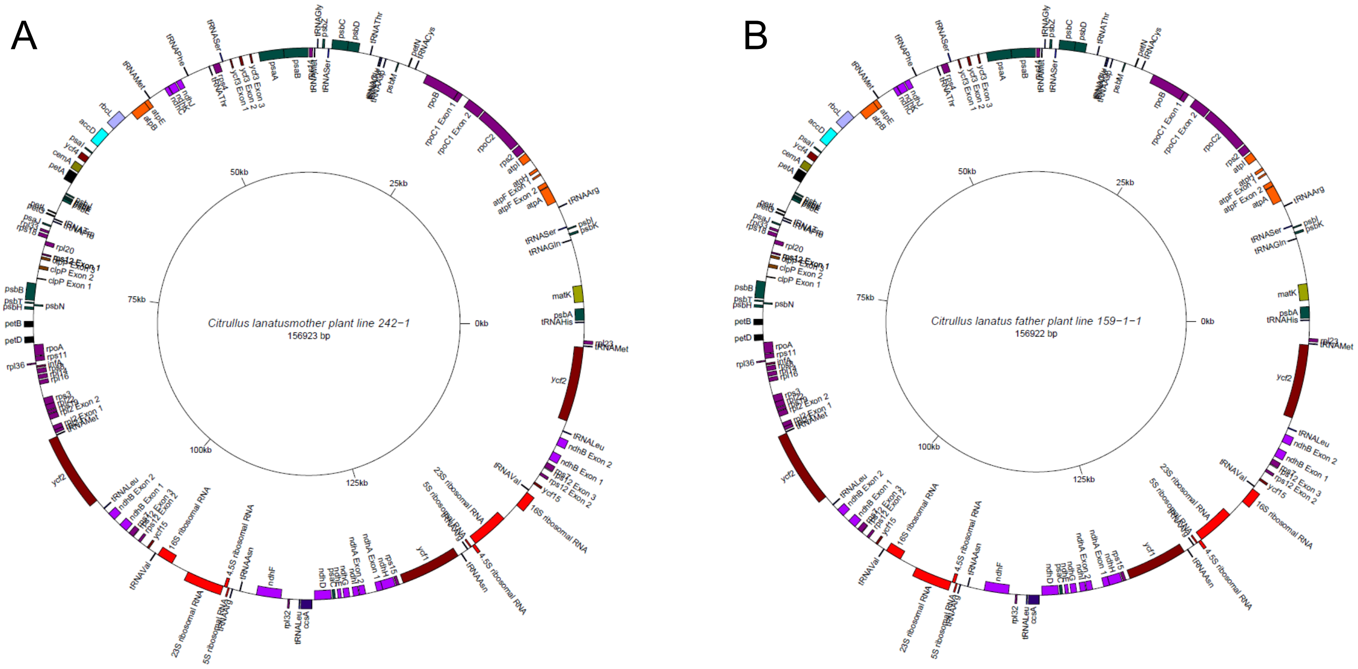

Supplementary Figure 2. Gene maps of chloroplast genomes of watermelon cultivars 242-1(A) and 159-1(B). Genes on the inside of the large circle are transcribed clockwise and those on the outside are transcribed counterclockwise. The genes are color-coded based on their functions. Dashed area represents the GC composition of the chloroplast genome.

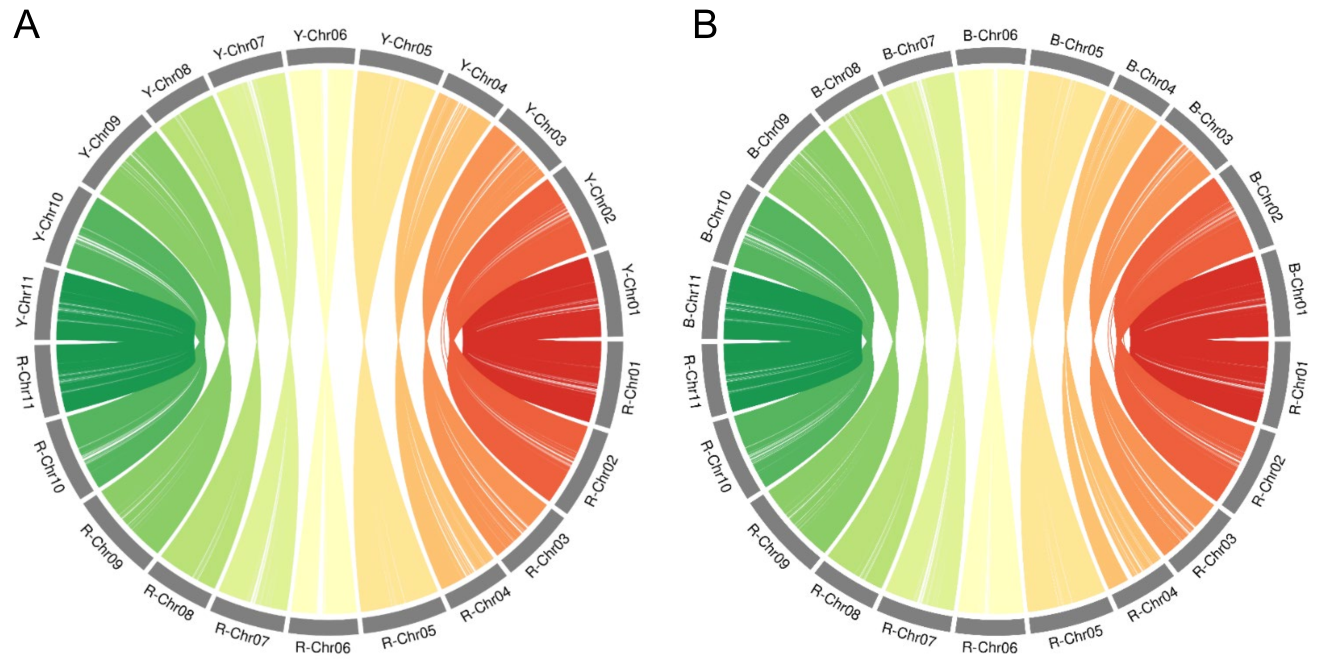

Supplementary Figure 3. Circos plot representation of synteny blocks. Circos plots between 97103 and 159-1 (A) and cv. 97103 and 242-1 (B).

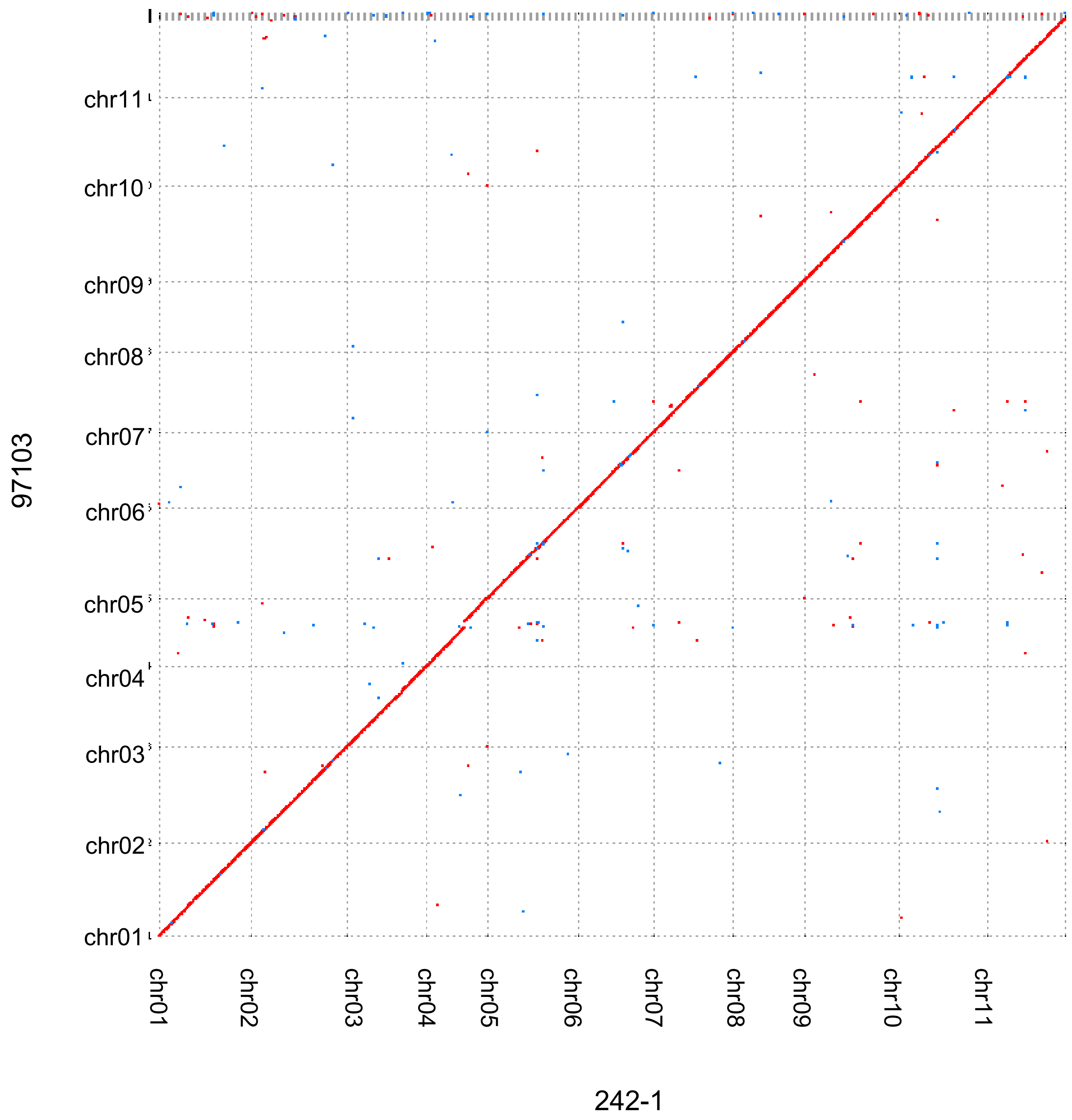

Supplementary Figure 4. Whole-genome nucleotide alignment of the 97103 and 242-1 genomes. MUMmer dot-plot displaying stretches of conserved sequence between the genomes of 97103 and 242-1 as lines with slope=1. Red lines represent sequence aligning in the same direction while blue lines represent inversions.

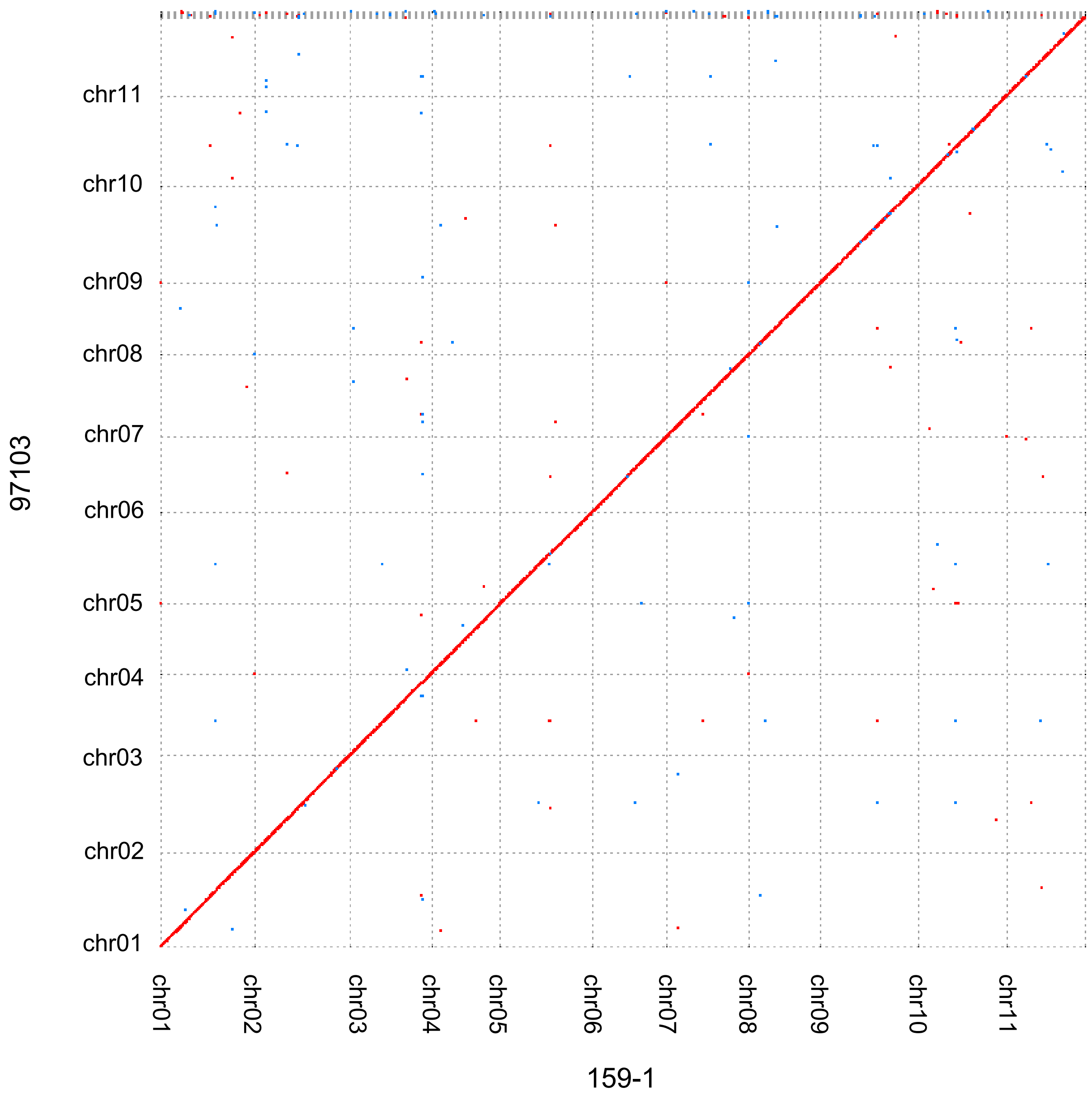

Supplementary Figure 5. Whole-genome nucleotide alignment of the 97103 and 159-1 genomes. MUMmer dot-plot displaying stretches of conserved sequence between the genomes of 97103 and 159-1 as lines with slope=1. Red lines represent sequence aligning in the same direction while blue lines represent inversions.

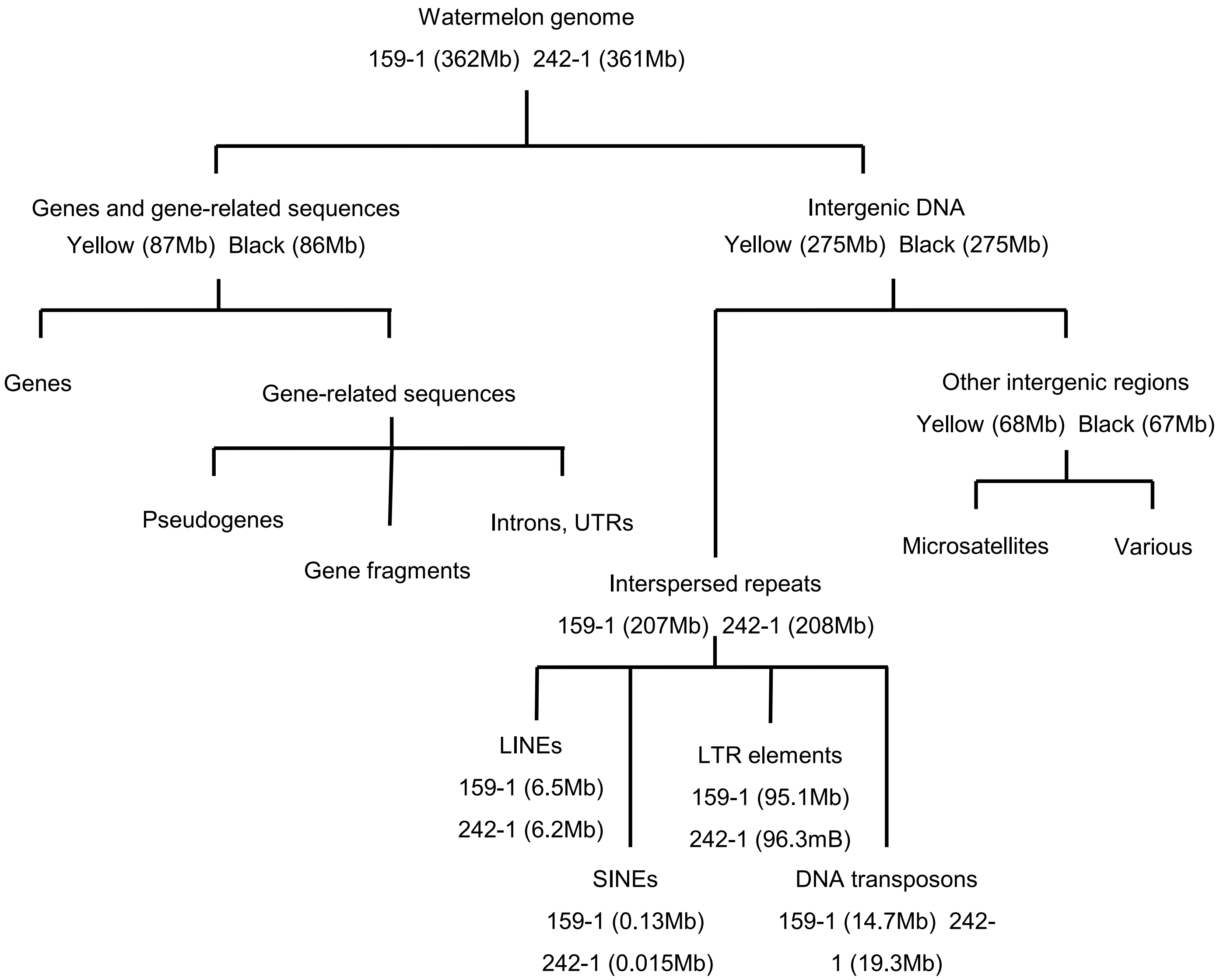

Supplementary Figure S6. Overview of watermelon genome 242-1 and 159-1.

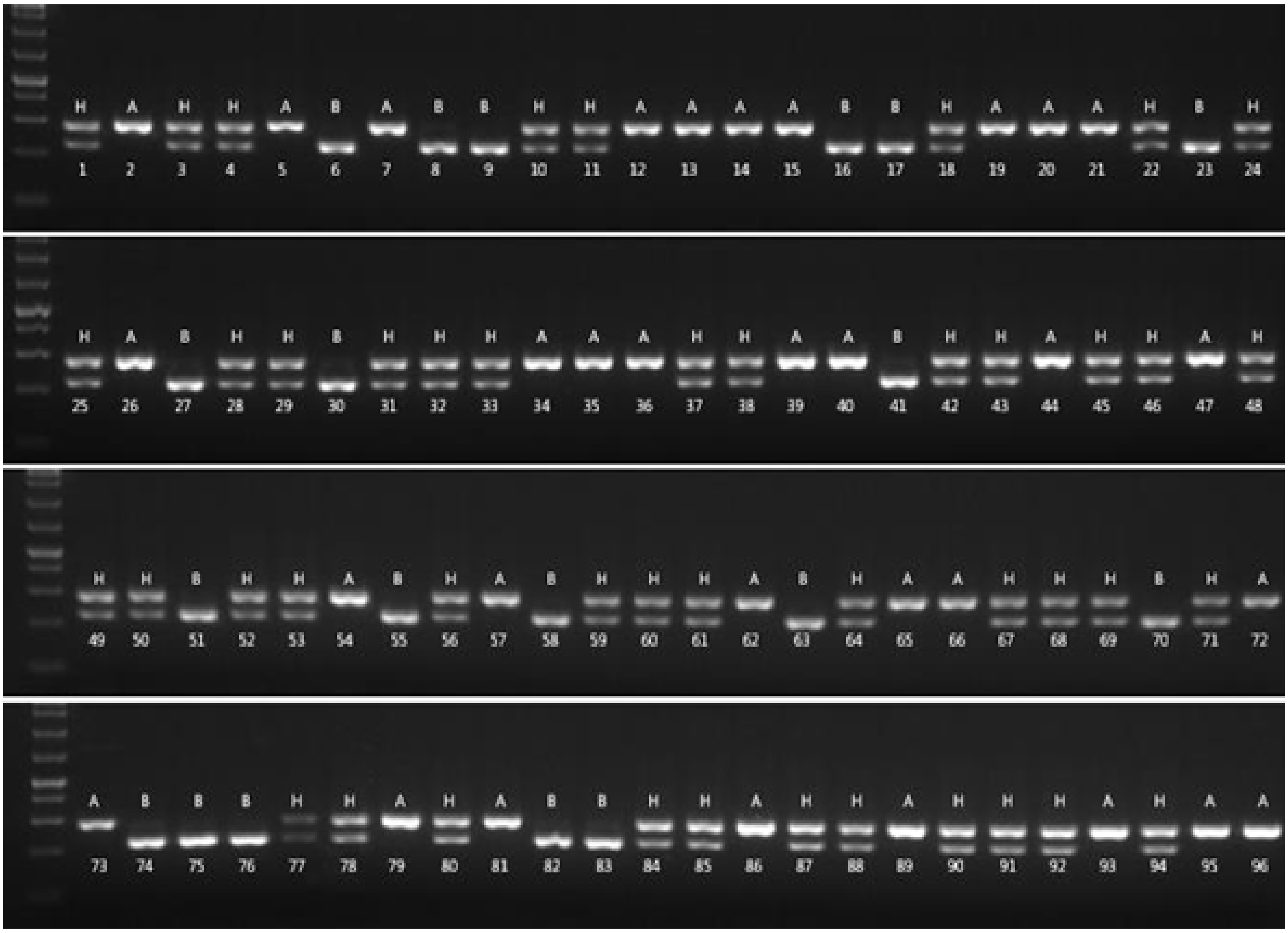

Supplementary Figure S7. The genotypes of the F2 plants at the Clg-InDel-27 InDel marker and verification of heteroduplexes. A, homozygous in maternal (242-1) genome sequence; B, homozygous in paternal (159-1) genome sequence; H, heterozygous genotype (F1 type).

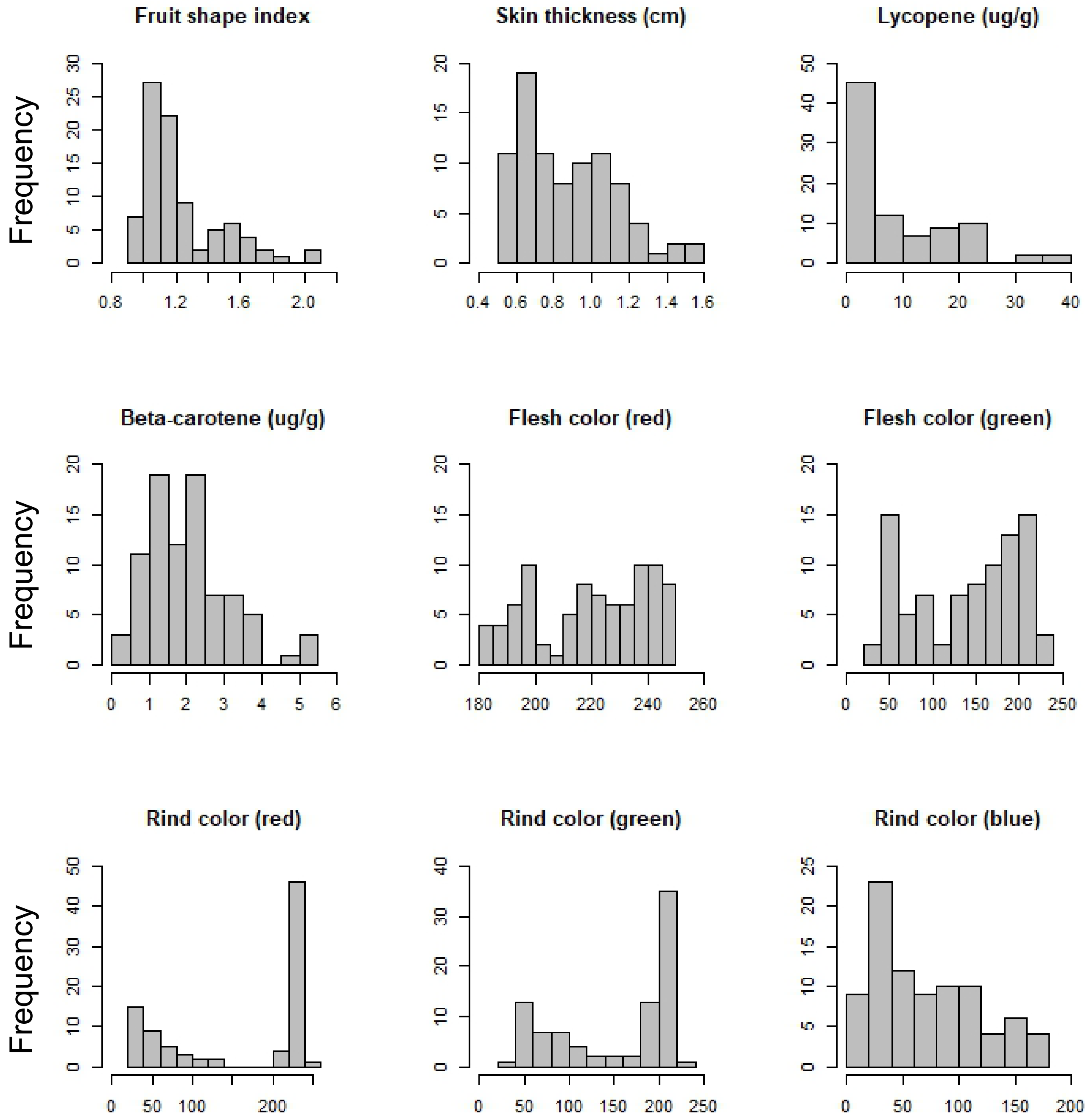

Supplementary Figure S8. Histogram for fruit quality-related traits of F2 watermelon plants.

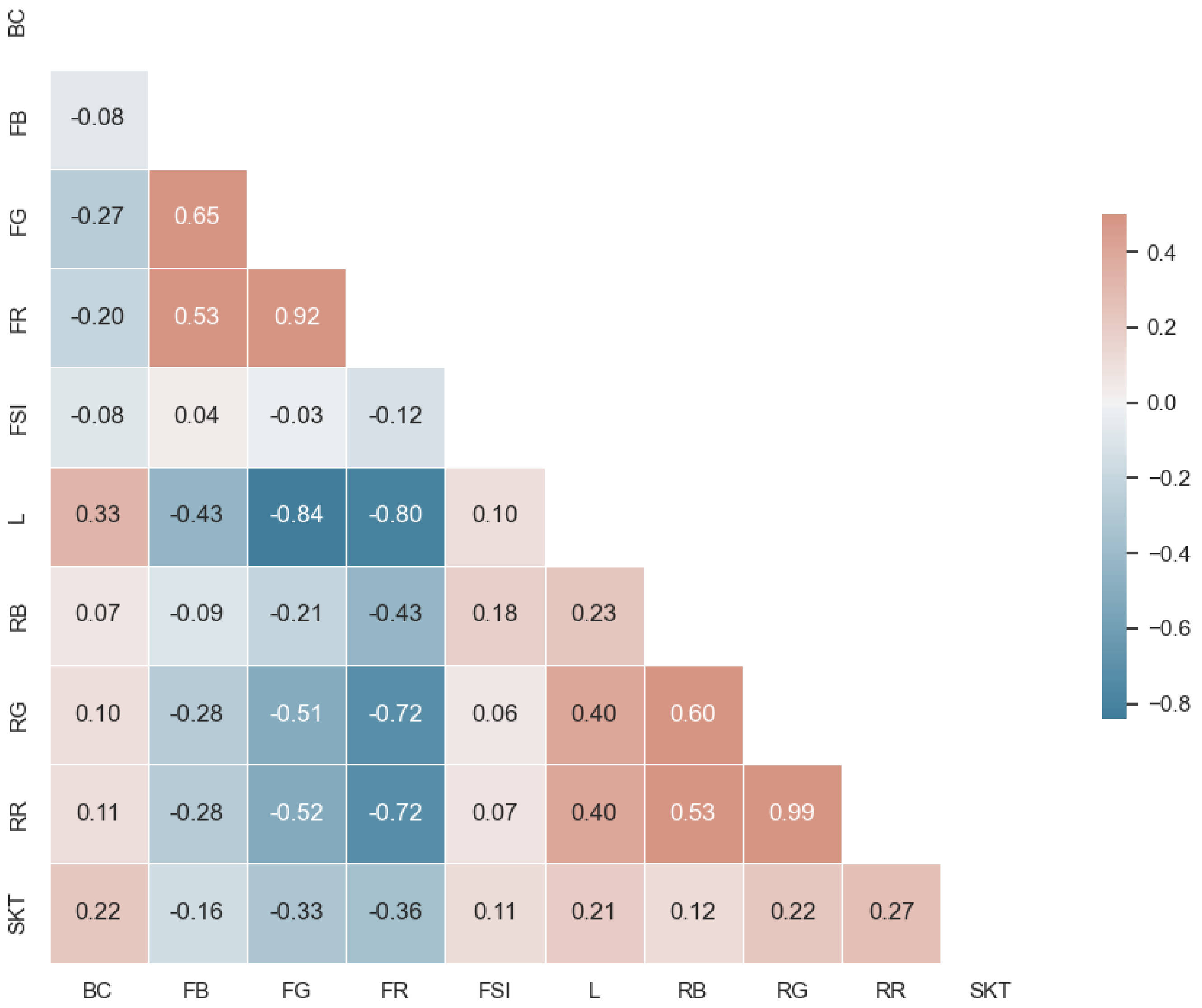

Supplementary Figure S9. Pairwise Pearson correlation matrix of the phenotypic traits. Red indicates positive correlation, and blue indicates negative correlation. BC: beta carotene, L: lycopene, FR: flesh color red, FG: flesh color green, FB: flesh color blue, FSI: fruit shape index, SKT: skin thickness, RR: rind color red, RG: rind color green, RB: rind color blue.

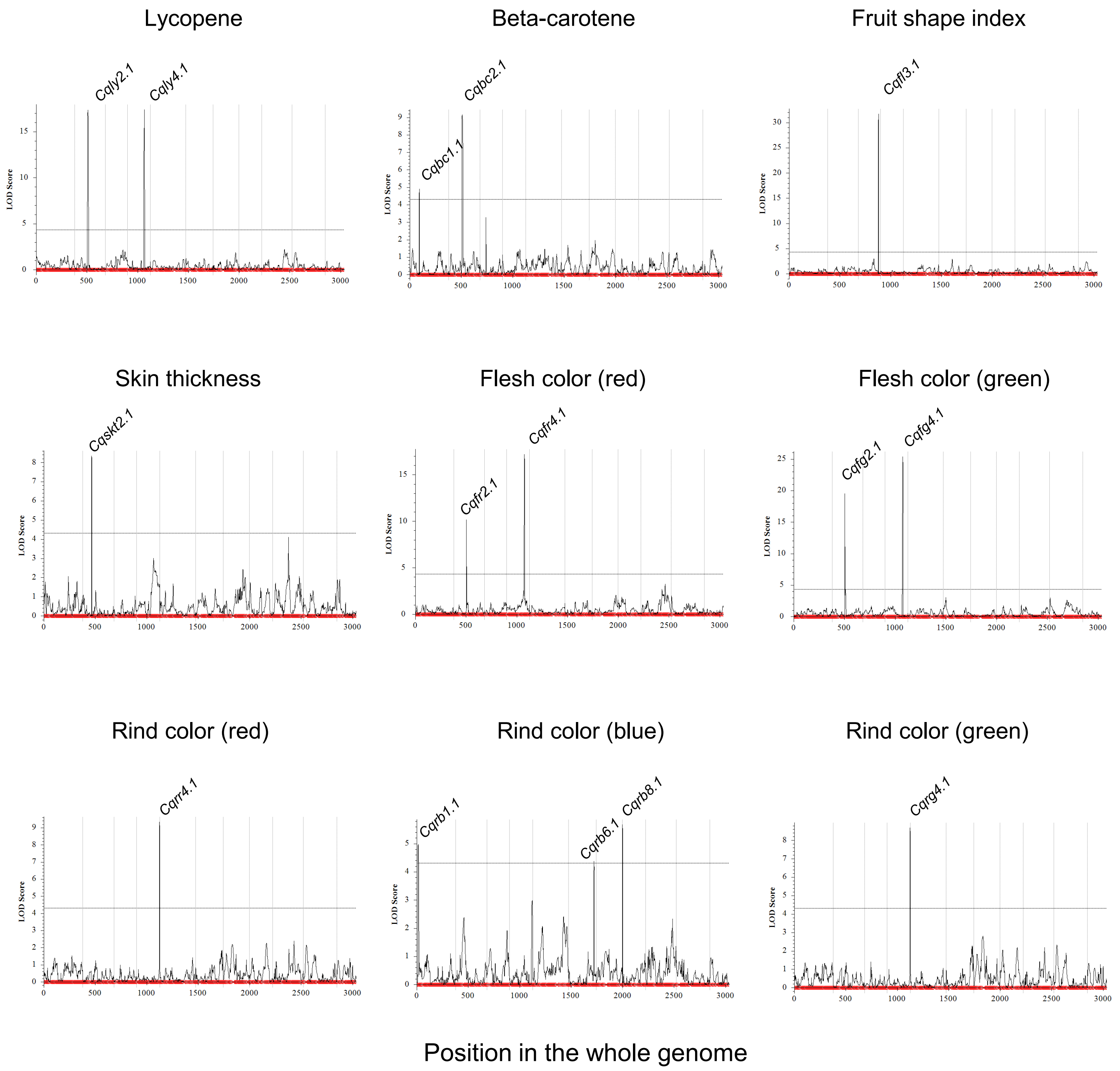

Supplementary Figure S10. Genomic location of QTLs for watermelon quality-related traits. Red lines in the bellow represent the position of the markers. LOD threshold = 4.3
